# The GoodHope Exercise and Rehabilitation (GEAR) Program for People With Ehlers-Danlos Syndromes and Generalized Hypermobility Spectrum Disorders

**DOI:** 10.3389/fresc.2021.769792

**Published:** 2021-11-08

**Authors:** Nimish Mittal, Daniel Santa Mina, Stephanie Buryk-Iggers, Laura Lopez-Hernandez, Laura Hussey, Alyssa Franzese, Joel Katz, Camille Laflamme, Laura McGillis, Lianne McLean, Maxim Rachinsky, Dmitry Rozenberg, Maxwell Slepian, Aliza Weinrib, Hance Clarke

**Affiliations:** ^1^GoodHope Ehlers Danlos Syndrome Clinic, Toronto General Hospital, University Health Network, Toronto, ON, Canada; ^2^Division of Physical Medicine and Rehabilitation, Temerty Faculty of Medicine, University of Toronto, Toronto, ON, Canada; ^3^Faculty of Kinesiology and Physical Education, University of Toronto, Toronto, ON, Canada; ^4^Department of Anesthesiology and Pain Management, Toronto General Hospital, University Health Network, Toronto, ON, Canada; ^5^Department of Psychology, Faculty of Health, York University, Toronto, ON, Canada; ^6^Department of Anesthesiology and Pain Medicine, Temerty Faculty of Medicine, University of Toronto, Toronto, ON, Canada; ^7^Division of Respirology, Temerty Faculty of Medicine, Toronto General Hospital Research Institute, University Health Network, Toronto, ON, Canada

**Keywords:** Ehlers-Danlos Syndrome, exercise, rehabilitation, models of care, Generalized Hypermobility Spectrum Disorder

## Abstract

**Introduction:** The Ehlers-Danlos Syndromes (EDS) and Generalized Hypermobility Spectrum Disorders (G-HSD) comprise a heterogeneous group of genetic disorders of abnormal synthesis and/or maturation of collagen and other matricellular proteins. EDS is commonly characterized by manifestations such as multi joint hypermobility that can lead to musculoskeletal pains, subluxations and dislocations, fragile skin, organ dysfunction, and chronic significant diffuse pain with fatigue, deconditioning eventuating to poor quality of life. Evidence suggests exercise and rehabilitation interventions may ameliorate symptoms of unstable joints, recurrent subluxations/dislocations, and chronic widespread musculoskeletal pain. To date, there have only been a few reports describing exercise and rehabilitation care strategies for people with EDS.

**Methods:** In this manuscript, we describe the GoodHope Exercise and Rehabilitation (GEAR) program, its overarching principles, as well as the program development and delivery model. The GEAR program aims to decrease functional impairment, reduce pain, increase confidence in symptom self-management, and provide a community of support for people with EDS/G-HSD. To achieve these goals, we detail the model of care that includes exercise and rehabilitation therapy, education for self-management, and support accessing relevant community resources.

**Strengths and Limitations of the Study:** GEAR represents a novel exercise and rehabilitation care model for people with G-HSD and various clinical EDS subtypes, beyond the commonly included hEDS subtype. Systematic collection of data via validated measurements is ongoing and will guide the refinement of GEAR and support the development of emerging exercise and rehabilitation programs for people with EDS.

## Introduction

The Ehlers-Danlos Syndromes (EDS) and Generalized Hypermobility Spectrum Disorders (G-HSD) are overarching terms for a heterogeneous group of genetic connective tissue disorders (1, 2). EDS/G-HSD predominantly affect the musculoskeletal, gastrointestinal, and cardiovascular systems and are characterized by the abnormal synthesis and/or maturation of collagen and matricellular proteins in the body (3, 4). The symptoms typically manifest as fragile skin, organ dysfunction, significant diffuse pain (2), and hypermobility that can lead to recurrent joint dislocations and other injuries (5–7). Of the 13 identified types of EDS, 12 can be confirmed by molecular analyses allowing for precise diagnosis and clinical direction on inheritance patterns, and can guide approaches to management. The most common EDS subtype, known as hypermobile EDS (hEDS), has an unidentifiable genetic profile. Thus, identification of this subtype relies on clinical presentation using objective 2017 EDS criteria including specific systematic manifestations of connective tissue disorder and joint hypermobility (1). The spectrum of joint hypermobility extends from asymptomatic joint laxity to generalized joint hypermobility. Individuals that exhibit generalized joint hypermobility (>4 or 5 joints [based on age]) and *do not* have a clinical presentation consistent with other heritable disease—including hEDS—are diagnosed with G-HSD (2). For consistency in this paper, all references to EDS or G-HSD will collectively be referred to as EDS/G-HSD.

Individuals with EDS/G-HSD can experience a unique set of physical sequelae, most commonly painful and/or unstable joints, chronic widespread musculoskeletal pain, and a history of recurrent joint subluxations and dislocations (8). For example, De Coster et al. (9) observed that dysfunction of the temporomandibular joint is reported in >70% of hEDS patients. Stanitski et al. (6) observed that 72% of hEDS patients reported joint dislocation −26% of which were hip dislocations. Additionally, Ainsworth et al. (10) observed dislocation of the shoulder and knee joints in 63 and 57% of hEDS patients, respectively. In light of the chronic pain and high risk of musculoskeletal injury, patients with EDS/G-HSD are prone to kinesiophobia [fear of movement and (re) injury] and behavioral responses that limit their involvement in essential and recreational activities (11). The resultant sedentary lifestyle may further exacerbate impairments, deconditioning, and compromise quality of life, while increasing the risk of cardiometabolic disease (12).

An often-proposed management strategy for clinical and functional manifestations of EDS/G-HSD is therapeutic exercise (13, 14). Therapeutic exercise is defined as a planned performance of physical movement, postures, or activities intended to provide patients with the means to remediate or prevent impairments of functions and structures; and in doing so, optimize overall health, fitness, or sense of well-being (15). Exercise in people with EDS/G-HSD has generally employed locoregional exercises that target isolated instability or impairment, such as knee proprioception (16), inspiratory muscle strength (17), and spinal stabilization (18). General conditioning-based exercise to ameliorate poor cardiorespiratory fitness and musculoskeletal strength that occur secondary to physical inactivity has also been highlighted in the literature as an important self-management strategy for symptom management (19, 20). Accordingly, exercise and rehabilitation are recommended in comprehensive EDS/G-HSD care models (18–20).

In this manuscript, we describe the GoodHope Exercise and Rehabilitation (GEAR) program. The mission of GEAR is to improve the physical function and psychosocial well-being of people with EDS/G-HSD through exercise and rehabilitation, education for self-management, and supported engagement in additional community resources. Opportunities for related research are also discussed in an effort to advance this nascent literature.

## Methods

GEAR is a part of the GoodHope EDS Clinic that is situated in the Toronto General Hospital in Toronto, Ontario, Canada (details of the GoodHope EDS clinic are provided elsewhere) (21). GEAR is delivered in an exercise and rehabilitation facility of ~1,200 square feet where assessments, exercise and rehabilitation therapy, and consultations related to education and community resources are conducted. The facility includes exercise and rehabilitation equipment, clinician workstations, and a patient waiting and changing area. GEAR is delivered by physiotherapists and kinesiologists (hereafter referred to as GEAR clinicians). This protocol was informed by relevant literature (15, 19, 20, 22) and designed by an inter-professional healthcare team, including kinesiologists, nurses, physicians, physiotherapists, and psychologists—each with experience specific to EDS/G-HSD or related injuries and chronic conditions. The program design was guided by expertise in the management of functional manifestations of EDS, implementation science, integrated research, home-based exercise, and psychosocial influences on treatment.

### Eligibility and Referral to GEAR

GoodHope EDS Clinic patients requiring exercise and rehabilitation care are directly referred to GEAR. Eligibility criteria for GEAR is determined in consultation with the patient and related to their overall function and symptom burden, interest and willingness to participate in exercise and rehabilitation, and ability to attend GEAR appointments. All referrals include a GoodHope EDS Impairment and Interference Scale (GEIIS) rating (23). The GEIIS is a brief, check-list style, medical screening tool that provides a general indication of a patient's physical function and impairment to advise appointment planning. Patients with a GEIIS rating of 4 (“Unable to perform all self-care activities; not ambulatory”) are not eligible for GEAR.

### GEAR Programming/Interventions

**Figure 1:**
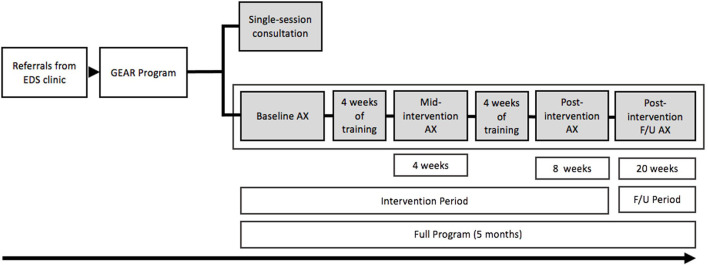
Depiction of the GEAR program and the full program time line of the intervention, assessments (AX), and follow up (F/U).

GEAR is delivered via three integrated service components: (i) individually tailored, home-based exercise and rehabilitation therapy; (ii) education on self-management for localized and systemic EDS/G-HSD symptoms; and (iii) connections with community-based services. Patient care plans are divided into two streams, an intervention stream and a consultation stream, summarized in Figure 1.

#### Intervention Stream

The GEAR intervention is initiated with a 2-h intake assessment with a GEAR clinician and includes a series of postural and functional tests to help identify symptomatic body regions and aberrant or dysfunctional movement patterns. It also includes a comprehensive patient interview focused on functional capacity, activities of daily living, pain (instigators and relievers), occupational and recreational physical activity, and general goals for physical and psychosocial wellness. Physical measures of function and pain are employed as needed and include range of motion, strength and muscle activation, proprioception, neurodynamic tension, balance, and aerobic endurance which collectively support the development of an 8-week care plan. Although exercise interventions have been observed to be effective on physical outcomes in as little as 4 to 6 weeks for individuals with EDS/G-HSD (16, 20, 22), To and Alexander (24) observed that the relationship between the length of the intervention and strength-gains is linear. Therefore, to effect greater change on physical outcomes, GEAR programming is delivered over an 8-week time frame, and to further these gains, has incorporated patient self-management education to enhance long-term adherence (20).

The intake assessment informs the provision of an 8-week individualized conditioning and rehabilitation exercise prescription that can be completed at home or at an available community-based facility that the patient has access to. GEAR intervention participants are expected to return at week-4 (mid-point) and week-8 (post-intervention) for re-assessment and programming adaptation (as required; see Figure 1 for patient flow). Patients return at 12 weeks post-intervention to assess the maintenance of symptom management goals and behaviors and are subsequently discharged from GEAR. If patients require further assistance with their GEAR activities or need assistance with transitioning care to a community health professional, they may book additional follow-up appointments (via telephone or in-person) for up to six months post-discharge. If patients require assistance beyond the six-month post-discharge timeframe, they require a re-referral from the GoodHope EDS Clinic. Patients may not complete the GEAR intervention multiple times; however, at the discretion of GoodHope EDS clinic staff, patients can be re-referred for a GEAR consultation (described below) to address new or evolved exercise and rehabilitation needs. The following sections detail the therapeutic objectives and strategies within each component of GEAR.

#### Exercise and Rehabilitation Therapy

Informed by the intake assessment, therapeutic and conditioning exercises are prescribed to reduce symptom burden and improve functional capacity. Initial exercise prescriptions consist of general aerobic exercise recommendations and 3–5 neuromuscular and/or resistance training exercises. Aerobic exercises are typically focused on increasing endurance and overall physical activity volume to meet the Canada's physical activity guidelines for adults (25). To ensure patient safety, patients undergo cardio-pulmonary screening with resting (sitting and standing) blood pressure, resting heart rate, and arterial oxygen saturation level before engaging in or receiving a prescription for aerobic activity. The resistance and neuromuscular exercises follow general guidelines such as: progressions from closed kinetic chain to open kinetic chain, proximal to distal, non-weight bearing to weight bearing positions, mid-range isometrics to through-range activation, bilateral to unilateral, and short to long lever. Additionally, these exercises work to challenge the limits of stability by reducing base of support, and introducing unstable surfaces or cognitive tasks. Patients are encouraged to participate in gentle dynamic movements and range of motion exercises as warmup activities prior to proprioceptive and strengthening exercises in order to reduce pain and risk of injury (26). These exercises are reviewed and adapted or expanded upon as tolerated (i.e., additional exercises added to the exercise prescription) during their 4- and 8-week assessments. Each prescription is tailored to the patient's EDS/G-HSD symptoms and may involve adapted exercises to accommodate joint hypermobility, orthostatic intolerance, gastrointestinal symptoms, multiple joint/organ symptom involvement, and localized or diffuse pain.

Exercise prescriptions are provided using the conventional frequency, intensity, time, and type (FITT) parameters for exercise volume using the modalities described above. Patients have an opportunity to practice the exercises before they leave their appointments and are educated on how to use a modified Rating of Perceived Exertion (RPE) Scale to monitor aerobic, neuromuscular, and resistance-based exercises (27). GEAR uses the modified RPE scale measuring 0–10, in which *easy* measures at 0–2, *moderate* at 3, *somewhat hard* at 4, *hard* at 5–6, and *extremely hard* at 7–10. Neuromotor exercises are typically prescribed in the 4–6/10 range (*somewhat hard* to *hard*), and resistance-based and aerobic exercises are prescribed in the 3–6/10 range (*moderate* to *hard*), and all prescriptions are progressed as tolerated. In addition to RPE, additional indicators of exertion are instructed, such as the breath-sound check and talk test. Additional factors to RPE are considered when prescribing exercise including mood states, baseline pain, environmental conditions (i.e., access to equipment or positional preferences), exercise mode, and age. Table 1 provides the GEAR program guidelines for exercise prescriptions and sample exercises are provided in Table 2.

**Table 1:** GEAR's exercise and rehabilitation guidelines for prescribing regimes to patients.

**Exercise type**	**Exercise and rehabilitation**
Aerobic	• F−4–7 days/week • I—RPE of 3–6/10 (moderate to vigorous) • T−10-30 minutes/session (150 min/week) • T—Walking (progress or regress based on symptoms or patient preference) **Notes**: • Duration, intensity and type of aerobic exercise will be decreased if onset of orthostatic symptoms occur in more upright postures • When able, incorporate a 5–10 min warm up/cool down with RPE 1–2 • See “Aerobic Training Protocols” for session specific details/guidance
Neuromuscular stabilization	• F—Daily or at least 5 days a week (there are no guidelines on neuromotor exercise intensity for this age group—this recommendation is based on the expectation that frequent practice will result in improved neuromuscular connections, movement patterning and proprioception). • I- RPE 3–6/10 (there are no guidelines on neuromotor exercise intensity—we are replicating the guidelines used to determine intensity for aerobic and resistance exercise) • T−3–6 exercises, depending on the patient's efficiency with neuromuscular control • T—See below for operational definition of Neuromuscular Exercise. **Notes**: • Neuromuscular exercise: to improve muscle control and stability of the joint, which leads to reduction in symptoms and improved quality of life • Neuromuscular exercises are taught and trained prior to the introduction of resistance-based exercises to ensure effective muscle activation, joint stabilization and posture prior to increasing demand on the patient • Patients are quickly transitioned to resistance-based exercises as they develop adequate activity and postural control involving problematic muscle groups • See “Table 2” for session specific details
Resistance	• F−2-4 days/week • I—RPE 3–6/10 • T−3–6 exercises, 1–3 sets/exercise, 8–15 repetitions/set (10–15 min/session) • T—Start in more supportive postures (lying, seated) using body weight or light resistance and progress to more demanding postures with greater resistance (bands, weights) **Notes**: • Use neuromuscular exercise principles to maintain good posture and proprioception to avoid aberrant movement patterns (i.e., joint hyperextension) • Monitor for orthostatic symptoms during exercise (e.g., presyncope) • Organize program to avoid multiple transitions from lying, seated or standing • See “Table 2” for more session specific details
Stretching and relaxation	• Encourage and/or support dynamic movements/range of motion exercises within normative values to reduce pain and as a warmup prior to aerobic/strengthening/neuromotor exercises
Balance and proprioception	• Encourage and/or support incorporation of activities that challenge alignment, body awareness and posture
**Modification principles**	
Progression of Exercise	Patients are shown progressions for each exercise and educated on when and how to progress based on RPE Patients can typically start a progression with RPE is consistently <3/10 and as long as any increase in baseline pain decreases within 2 h after ceasing the exercise • Patients are first encouraged to progress the number of repetitions within a set, then the number of sets, in order to build endurance before moving onto a new version of the exercise • Exercise difficulty is increased by changing level of resistance, patient position or a combination of both
Regression of exercise	Patients are shown regressions for each exercise and educated on when and how to regress based on RPE • Patients are encouraged to regress when RPE is > 6/10 or increased pain persists 2 h after ceasing the exercise (28) • Patients are also encouraged to regress to a less effortful version of their program following a flare or acute injury
General	• General progression guidelines go from closed kinetic chain to open kinetic chain, non-weight bearing to weight bearing positions, mid-range to through-range, bilateral to unilateral, short to long lever, reducing base of support, activities within base of support to challenging the limits of stability, introducing unstable surfaces or cognitive tasks • Furthermore, exercises can be modified by changing the number of reps/sets, changing positions or supportive equipment, intensity, number of rest breaks, or changing the exercise all together (progression/regression) • For patients with significant joint instability limiting participation, they are encouraged to wear splints/braces/external supports for the duration of the activity • Patients are encouraged to use prescribed gait aids for walking programs if needed

**Table 2:** Sample of GEAR's exercise and rehabilitation exercises.

	**Aerobic exercise**	
	Given its high functional application, every patient is encouraged to participate in daily walking. If walking over ground is too challenging, patients can try the following: • Walk on a treadmill (with upper extremity support) • Walk in water (hydrotherapy) Other exercises include: • Upright, recumbent or semi-recumbent exercise bike • Elliptical trainer • Swimming • Rowing For patients with low physical activity levels, low activity tolerance, or low upright tolerance; patients can perform any of the following seated activities: ^*^Note that seated activities should be repetitive/rhythmical in fashion, performed for at least 10 min, with an RPE of 3–6 (28) ° Marching ° Toe and heel tapping ° Reaching arms and legs to the side ° Arm ergometer ° Seated stepper/peddler ^*^High impact activities such as running, stair climbing, and those with stop and go actions are not recommended if limited by lower body arthritis (28)	
	**Neuromuscular exercise**	**Resistance exercise**
**Upper extremity**		
Neck	Deep Neck Flexor Activation with tactile and verbal cueing • Supine, blood pressure cuff/towel placed under the neck for feedback • Isometric holds avoiding accessory muscle use or breath holding Diaphragmatic Breathing Supine, Seated and Standing postural training awareness • Anterior, posterior and neutral pelvic tilt positions • Equal weight bearing through ischial tuberosities (ITs) in sitting (avoid sacral sitting) • Equal weight bearing through heel and ball of foot in standing • Thoracic extension If applicable, initiate Scapular Stabilizer Activation (see “Shoulder” Neuromuscular Exercises) If applicable, initiate Gaze Stability and Head-eye Coordination Exercises (as appropriate) Incorporate functional activities as able • Looking and walking	Deep Neck Flexor Activation with: • Supine/seated/standing ° Resisted cervical spine isometrics (with/without band/ball) ° Lower c-spine flexion with cranio-cervical flexion • Plank/modified plank ° Resisted c-spine lateral flexion isometrics ° Lower c-spine extension to neutral Scapular Stabilization Exercises as appropriate (see Shoulder Exercises) Core Exercises as appropriate (see Low Back Exercises)
Upper/Mid back/Ribs	Start with “Neck Neuromuscular Exercise” and Costal Breathing Re-education Diaphragmatic Breathing Supine, seated and standing posture awareness Rotation/Dissociation Exercise • Segmental rolling in supine • Thoracolumbar control in 4-point kneeling	Initiate Neck and Low Back Exercises (as appropriate) ^*^Note if patient demonstrates rib flaring or excessive t-spine extension, encourage upper abdominal activation, control and posture • Bed/Wall Angel Exercise • A, W, T, Y, I Formation Exercices Introduce Additional Scapular Stabilization Exercises (see Shoulder Exercises) Seated/Standing Trunk Rotation • Uniplanar or multiplanar (proprioceptive neuromuscular facilitation pattern) • With/without resistance band/ball
Shoulder	Start with “Neck Neuromuscular Exercise” Scapular Stabilizer Activation with tactile and verbal cueing • Identification of “Scapular Setting” position (retracted and depressed) • Serratus Anterior Activation	Scapular Stabilizer Activation with: • A, W, T, Y, I Exercices • Floor/Wall Angel Exercise • PNF Pattern Exercises with resistance band or pulleys – supine, seated, standing
	• Side lying, supine or kneeling/plank positions as appropriate • Rotator Cuff Activation • Side lying against resistance • Trapezius, rhomboids, teres major, latissimus dorsi activation Remind patient of Supine, Seated and Standing postural training awareness	•Scapular Protraction/Retraction ° Prone on elbows ° Serratus punch (band or free weight) ° Push-ups (wall, knees, full) ° High/Low/Bent over row with free weights • Rotator Cuff Exercises ° Isometric external rotation walk-outs ° Isotonic external rotation at 0 degrees and 90 degrees abduction +/- resistance band • Forward/Lateral raise to 90 degrees • Functional exercises as appropriate: ° Lifting, reaching overhead, ergonomics Introduce Neck and Shoulder Exercises as appropriate
Elbow	Start with Neck Neuromuscular Exercises and/or Shoulder Neuromuscular Exercises if appropriate Triceps Activation: • Identification of “neutral” elbow position (avoiding hyperextension or valgus stresses) ° Wall push up or wall high plank ° Backward propped sitting position (no vision) • Teach self-facilitation with tapping/towel if appropriate	Before performing elbow specific resistance exercises, ensure the patient has adequate neck and shoulder postural control and strength. Elbow Flexion/Extension: •Isometrics at different ranges • Isotonics with resistance band or dumbbell • Functional exercises as able: ° Triceps dip in chair or on bench ° Push up variations • Task specific exercises maintaining alignment: ° Lifting a bag, pushing/pulling a door, getting up from a chair etc. Elbow Pronation/Supination isometrics or isotonics with resistance band or dumbbell (if applicable) Wrist/Grip Activities if appropriate (see Wrist/Hands Exercises)
Wrist/Hands	Start with Neck, Shoulder and Elbow Neuromuscular Exercises where appropriate Determine the following: • Neutral Positions and avoiding hyperextension of: IP, MCP and radiocarpal/ulnocarpal positions. • Activation of: ° Lumbricals: “L Position” ° Finger Flexors/Extensors Introduce minimal weight bearing wrist extension maintaining alignment and activation • Weight bearing on a table with forward and backward weight shifting	^*^Ensure the patient has adequate neck, shoulder and elbow postural control and strength. If proximal control is impaired, have the patient in more supported when performing hand specific training activities. Wrist Flexion/Extension and Radial/Ulnar Deviation • Isometrics, concentrics, eccentrics with resistance band or free weight • Clockwise and counterclockwise towel wringing • Radial/Ulnar Deviation with a hammer/dumbbell pronation/supination holding onto a hammer/dumbbell Weight bearing wrist extension ° Against a ball on the wall, alphabets, circles ° 4-point weight shifts Grip Strengthening with Putty or Elastics Functional Exercises • Farmer carries • Cooking—transferring plates, cups, chopping/cutting • Typing and pushing buttons
**Lower extremity**		
Low Back	Core Activation with tactile and verbal cueing • Identify full range of motion by cueing anterior and posterior pelvic tilts • Identify neutral spine position • Transversus Abdominus activation with palpation medial to iliac crests • Practice grading TA activation—i.e., 100% and 50% of Maximum Voluntary Contraction determined. TA Activation with diaphragmatic breathing TA Activation with heel slides, fall outs, heel taps etc.	Transversus Abdominus Core Activation with: • Positional holds (Dead Bug/Table Top Position) • Dead Bug with arm/leg movements • Bird Dog (4-point position) with arm/leg movements • Seated Pelvic Clock • High/low planks, side planks, leg lifts Supine/Seated/Standing Trunk Rotation (see Upper/Mid Back Exercises)
Hips	Start with “Low Back” Neuromuscular exercises. • Gluteal setting in supine or crook lying position • Supine marching, fall outs, adductor squeezes with ball • Partial sit-to-stand from perched sitting position (high surface with tap) encouraging proper alignment and muscular patterning	Transversus Abdominus Core Activation with: • Hip Abduction (double crook or side lying +/- resistance band) • Glute Bridge • Hamstring Curl with exercise ball • Partial Sit-to-Stand from perched sitting position (high surface with tap)
		Functional Exercises • Full Sit-to-Stand/Squat +/- hover • Squatting to pick objects off the floor
Knees	Depending on posture, may be encouraged to start with “Low Back” Neuromuscular exercises. Isometric Quadriceps Activation • Place a rolled towel behind the knee in a supine or long sitting position for feedback • Teach self-facilitation/stimulation with tapping or a towel ^*^Evaluate quadriceps activation, evaluate patellar mobility with activation Quads Over Roll +/– holds Straight Leg Raise +/– holds Functional Exercises • Full Sit-to-Stand/Squat/Hover • Stairs negotiation practice • Gait re-training exercises ° Forward/backward walking Side stepping (+/– resistance band)	Transversus Abdominus Core Activation with: • Hamstring Curl on therapy ball • Partial Sit-to-Stand/Squat (see above) • Step Up with Tap Back (forward/lateral) • Sliding Lunges (backward and lateral)
Ankles/Feet	Foot Intrinsic Activation (“Short Foot” Exercise) • Start barefoot, in sitting (minimal WB position) • “Lift your toes off the ground to raise the arch of your foot. Now gently lower your toes, maintaining the arch of your foot” Progress length of holds, then to bilateral stance, then unilateral stance Functional Exercises • Tandem and Single Leg Stance practice • Gait re-training exercises (see above) • Heel/Toe/Tandem Walking • Dual tasking (i.e., walking and picking up an item off the floor)	Dorsiflexion/Plantar Flexion/Inversion/Eversion • Resisted isometrics (against ball/wall/resistance band) • Isotonics through range (+/– resistance band) Foot Intrinsic Activation with: • Standing on a compromised surface (foam, pillow) • Barefoot Heel Raises

* *Please note that exercises listed in the table above serve as a general guideline/framework for exercises provided to the patient and are dependent on which joint(s) the patient/clinician determine are problematic. This is not an inclusive list and are subject to changes and/or modifications*.

#### Self-Management Education

Basset's group found that patients' remissions and beliefs about their injuries or disorders can influence their level of rehabilitation adherence (23). Thus, individuals living with conditions such as EDS/G-HSD may perceive common symptoms such as chronic pain or reoccurring pain flare up episodes as barriers to exercise. GEAR patients receive self-management education (SME) in order to remain adherent to their prescribed exercise and rehabilitation program and engage in an active lifestyle for general health promotion, cope with episodic flare-ups of their pain, and reduce kinesiophobia. Progressive self-management strategies are discussed to support patient autonomy in establishing and adapting their treatment and health-related goals, as well as enhancing self-motivation and self-efficacy for long-term health behaviors (23).

GEAR SME focuses on the behavioral response to an acute pain flare-up which can include: employing pain-relieving positions; managing acute and recurrent subluxations; using mobility aids, braces or splints for joint support; using hot or cold modalities (e.g., compresses and packs); and modifying exercises to minimize or relieve pain. Second, energy conservation strategies are discussed in order to manage fatigue and to improve capacity for and adherence to physical activity. Finally, topics such as counter pressure maneuvers to manage orthostatic intolerance and long-term health behavior strategies, such as SMART goal setting (Specific, Measurable, Achievable, Realistic and Time-limited) approach (29) are used identify goals and monitor effectiveness of treatment.

#### Community Resource Engagement

Given the variable and recurrent nature of pain and impairment experienced by individuals with EDS/G-HSD, patients require hands-on therapy for acute symptom management, modification of a program, or aid in addressing the onset of a new symptom. As such, connecting with a community-based healthcare professional is key in providing ongoing exercise and rehabilitative care. Community connectedness is defined as belonging to a larger collective with whom an individual can establish mutually influential relationships, satisfy their needs, be rewarded through their affiliation, and build shared emotional connections (30, 31). It has been shown that community connectedness can lead to a positive impact on physical health outcomes with higher levels of connectedness being related to less pain, more energy, better physical functioning, and fewer limitations in those with chronic disease (32, 33). Further, maintenance of exercise regimes, especially in chronic disease populations, has been shown to significantly contribute to the preservation of functional benefits gained through short-term exercise programs (34, 35). Recognizing the impact of connectedness on health, the contribution of exercise maintenance, and the limited duration of the GEAR program (20 weeks including all standard follow-ups), the *community resource engagement* component aims to build a network of support for patients following their participation in the program. Patients are supported in this process through encouragement to develop relationships with a community exercise and rehabilitation professionals through the following supportive aids:

(i) Finding a local exercise and rehabilitation professional: Patients are guided through the process of finding a local clinician that is best suited for their individual rehabilitation and exercise-related conditions and/or goals. Patients are encouraged to request a clinician who has previous experiences treating people with EDS/G-HSD or an interest in learning about the sequelae that people with EDS/G-HSD commonly present with. Patients are also encouraged to seek clinicians that have specialization in specific areas of need common to the EDS/G-HSD population such as pelvic floor or TMJ dysfunction, acupuncture, or sport specific rehabilitation. Exercise and rehabilitation professionals that may be involved in community-based care include: physiotherapists, occupational therapists, orthotists, acupuncturists, massage therapists, kinesiologists, aquatic rehabilitation professionals, and personal trainers. These health professionals may be accessible at local fitness, community, wellness centers or private clinics. As (36) have observed, patients' experiences during treatment can significantly influence the outcomes of rehabilitation therapy, and thus patients are recommended to seek support from those who share similar interests and/or treatment philosophies.
(ii) Best practices while working with a community health professional: Patients are encouraged to employ several recommended practices when working with a community health professional, including setting SMART goals, which should be regularly revisited and measured in order to track changes. Patients are also encouraged to set a specific timeline and open discussion on plan of care (frequency and nature of sessions) to avoid early exhaustion of insurance benefits. Lastly, upon request, a summary of GEAR clinical notes and/or patient-specific recommendations can be shared with the local health professional.

#### Consultation Stream

Some GoodHope EDS Clinic patients who are appropriate but unable to participate in GEAR due to significant barriers to program participation, such as distance from the facility resulting in significant time or monetary costs associated with attending the program appointments or competing priorities that preclude engagement in routine exercise and rehabilitation. These patients may be referred for a single consultation session comprising generalized assessment and SME similar to those described in the baseline assessment in the GEAR intervention but is distilled to address only the top priorities of the patient's needs that can be supported within a single, two-h consultation appointment. Similar to the GEAR intervention, patients in this stream requiring further assistance are able to book additional follow-up telephone appointments with a GEAR clinician for up-to six months in efforts to help network-building with community-based services.

## Anticipated Results and Data Collection

Given the dearth of evidence to guide exercise and rehabilitation therapy for people with EDS/G-HSD, a core function of GEAR is to systematically collect data relevant to patient outcomes and program participation. Within GEAR, standardized data collection occurs at each assessment which are intended to provide insight for patient care as well as contribute to a data repository for potential research use in cross-sectional and longitudinal analyses. It is important to make the distinction that GEAR itself is not a study but a program that supports current and future studies. An institutional ethics board clearance for data collection has been approved for all studies pertaining to GEAR, as will all future studies. All the patients willing to participate in the research arm of GEAR are contacted by a research assistant to explain the study in detail and for consent. Only consenting individual's data will be used in comprehensive analysis for publications purposes. The specific data collected at each time point are described in Table 3. The functional tests outlined in Table 3 were selected based on the following characteristics: (i) requirement of minimal equipment and easily replicable, (ii) a short test duration to limit patient burden, (iii) accessible given the physical sequelae common to individuals with EDS/G-HSD; (iv) and together capture changes in upper extremity strength, lower extremity strength, cardiovascular capacity, and postural control and balance. Although these are robust as a collection of tests, it is recognized that additional tests are needed to capture the full potential effects of GEAR interventions. Additional measures may be employed to further inform care but may not be routinely collected for all patients, such as five times sit-to-stand test (37), gait assessment, and stair climb test. Beyond the functional test results, comprehensive charting is also recorded which describes each patient's physical assessment, their response to previous sessions, maintenance therapy, and any adverse effects. This systematic collection of validated measurements and charting will guide the refinement of GEAR and support the development of emerging exercise and rehabilitation programs for this underserved population.

**Table 3:** Clinical research measurements at given assessment periods of GEAR.

	**Baseline**	**4 week**	**8 week**	**20 week**
**Clinician measurements**				
Height (cm)	x			
Weight (kg)	x		x	x
Waist circumference (cm)	x		x	x
Six-minute Walk Test (6MWT)	x		x	x
Timed up and go test	x	x	x	x
Tandem balance	x	x	x	x
Single-leg balance	x	x	x	x
Grip strength (kg)	x	x	x	x
**Patient-reported outcomes**				
Godin leisure-time exercise	x	x	x	x
Lower extremity functional scale	x	x	x	x
Bristol impact of hypermobility	x	x	x	x

*Exercise testing considerations: for balance testing, additional care to be taken for those experiencing severe orthostatic symptoms; if patient is unable to perform tandem balance test, avoid 1-leg stance test. For aerobic fitness testing, 6MWT Test to be avoided for patients experiencing moderate to severe orthostatic symptoms; use of mobility aids during walking tests are encouraged; orthostatic symptoms as well as joint pain will be monitored closely during the 6MWT*.

## Discussion

In this manuscript, we describe a model of exercise and rehabilitation care for people with EDS/G-HSD—an area of clinical care that remains largely underreported. This is likely due to the limited evidence describing the use and benefit of such interventions in ameliorating EDS/G-HSD related symptoms. In a recent systematic review, Palmer et al. (38) identified only 11 studies that explored the effect of exercise interventions in people with EDS/G-HSD showing that they were beneficial for various physical and psychological outcomes, such as improved muscle strengthening, proprioceptive acuity, and quality of life (38). In light of the emerging evidence and clinical rationale for symptom-management and health promoting therapy, the GEAR program was developed as a core component of comprehensive EDS/G-HSD care in the GoodHope EDS clinic.

GEAR has several strengths to highlight: (i) program components have been developed based on an integrated research design, (ii) the program is delivered by an interprofessional clinical team including physiotherapists, kinesiologists, and physicians—each holding experience specific to EDS/G-HSD, (iii) it is a multi-pronged program including exercise and rehabilitation treatment, SME, and community resource engagement support; (iv) the program is offered to people with G-HSD and various clinical EDS subtypes—beyond the commonly included hEDS subtype, (v) treatment plans are individually tailored, (vi) focus is given to both acute and long-term outcomes; (vii) programming is delivered in a dedicated fully-equipped facility, and (viii) program participation is free of charge.

We also note challenges that GEAR has faced, namely its location within an urban hospital that poses attendance related barriers (e.g., commute time, cost of parking, and public transit can be a burden for some). GEAR is also only available to patients during regular business hours, which provides a barrier to those who have limited time available throughout the working day. Related to its setting, we also note that our program and its development may not be generalizable to community hospitals or other health care systems with different organizational barriers and resource constraints. Nevertheless, iterations of this program are likely possible in academic hospitals with a strong research program and sufficient financial support. An important aim of the GEAR program is to establish connections with community partners, to improve accessibility, and in turn, increase program reach. Importantly, the absence of high-quality research in this field is also noteworthy. This gap in the literature justifies an investment in research to optimally support evidence-based care. Through the combination of available evidence and interdisciplinary expert contributions, this initial iteration of GEAR has been established and will remain responsive to evolutions in the research literature.

Future growth of GEAR is multifaceted and involves both improving on the delivery of the program, as well as extending care to additional subgroups. In terms of program structure, GEAR will be implementing electronic data capture for both clinical and research data entry with the aim of improving the speed of certain processes, and the turnaround time for accessibility and updating data in real time. The program is also exploring social support interventions, such as group exercise classes to enhance participant adherence to positive behavioral changes. Lastly, with increasing access to the Internet and the ongoing development of technological platforms, remotely delivered Internet-based treatment approaches are being explored as a future alternative and/or complimentary support for the delivery of GEAR interventions. GEAR also aims to extend care as a potential management strategy beyond EDS/G-HSD, to include leading co-morbidities of EDS/G-HSD including Postural Orthostatic Tachycardia Syndrome (POTS) and Craniocervical Junction Instability (CCJI). Autonomic dysfunction, also known as dysautonomia, is a long-recognized complication of EDS/G-HSD and has symptoms reported at high rates (up to 78%) (39, 40), with POTS as the most prevalent autonomic profile (41). Aerobic reconditioning therapy through exercise has not yet been employed as treatment for POTS in patients with EDS/G-HSD, despite its known efficacy in improving or resolving POTS in most patients without EDS/G-HSD (28, 42). For example, George et al. demonstrated that in an international POTS patient registry, the vast majority of patients (71%) who completed 3 months of exercise training no longer qualified for POTS criteria and were thus effectively in remission (42). GEAR aims to extend these findings to the EDS/G-HSD population in an effort to reduce POTS-related symptoms, and thus potentially improve functional capacity and quality of life of those with EDS/G-HSD.

Additionally, individuals with EDS/G-HSD frequently present with headache, neck pain, and decreased cervical muscle strength. These symptoms have been hypothesized to be related to a potential CCJI (43). The craniocervical junction, a component of the spine, is made up exclusively of synovial joints and ligaments that allow for wide head and neck movements. It is especially vulnerable to the laxity of ligaments experienced by individuals with connective tissue disorders, such as EDS/G-HSD (44). Management of the symptoms related to CCJI are not well-established, and often when instability cannot be detected using current radiographic measures, the treatment of such symptoms remains limited. It has been suggested that non-surgical treatment should be employed with the goal of decreasing stresses on the involved spinal segments and to enhance the function of spinal stabilizing subsystems (45). Strengthening exercises have been shown to enhance the function of these subsystems (46). While the most effective exercises for this purpose have yet to be identified, exercises that focus on controlled motion and proprioception could address elements of stabilization, and if attained, prevent degenerative changes and the need for surgery (45). Further, exercise and rehabilitation therapy has been shown to improve pain and function of lumbar spine in people with EDS/G-HSD (18). It is hypothesized that such therapy has the potential to produce similar results for CCJI, and as such, GEAR aims to offer it as a form of treatment for the EDS/G-HSD-related complication of CCJI (45).

## Conclusions

Amid the plethora of exercise and rehabilitation intervention studies for chronic diseases, there remains a significant gap in its effect on people with EDS/G-HSD. The goal of GEAR is to transform the management of EDS/G-HSD through a multi-pronged exercise and rehabilitation treatment approach that has been designed using integrated research and interdisciplinary expert contributions. Through this approach, GEAR aims to decrease functional impairment, reduce pain, increase confidence in symptom self-management, and provide a community of support. Future clinical and research initiatives of GEAR will support the development, improvement, and accessibility of exercise and rehabilitation therapy programs for the greater EDS/G-HSD population.

## Data Availability Statement

The original contributions presented in the study are included in the article/supplementary material, further inquiries can be directed to the corresponding author/s.

## Author Contributions

NM, DSM, and HC designed the protocol and coordinated the project. NM, AF, LH, LL-H, and LMcG informed program design and delivery. DSM and SB-I informed data collection design. DSM, SB-I, and AF drafted the manuscript. HC is the guarantor. All authors critically reviewed and approved the manuscript.

## Funding

The GEAR Program is supported by the GoodHope Foundation and by the UHN Foundation.

## Conflict of Interest

The authors declare that the research was conducted in the absence of any commercial or financial relationships that could be construed as a potential conflict of interest.

## Publisher's Note

All claims expressed in this article are solely those of the authors and do not necessarily represent those of their affiliated organizations, or those of the publisher, the editors and the reviewers. Any product that may be evaluated in this article, or claim that may be made by its manufacturer, is not guaranteed or endorsed by the publisher.

## Abbreviations

6MWT: Six-minute Walk Test
CCJI: craniocervical junction instability
EDS: Ehlers Danlos Syndromes
GEAR: GoodHope exercise and rehabilitation
G-HSD: generalized hypermobility spectrum disorder
HCP: health care professionals
hEDS: hypermobile EDS
POTS: Postural Orthostatic Tachycardia Syndrome
PT: physiotherapist
RPE: Rating of Perceived Exertion
SMART: Specific, Measurable, Achievable, Realistic and Time-limited
SME: self-management education.
